# A Blood Test for the Diagnosis of Multiple Sclerosis

**DOI:** 10.3390/ijms25031696

**Published:** 2024-01-30

**Authors:** Paola Giuliano, Giuliana La Rosa, Serena Capozzi, Emanuele Cassano, Simona Damiano, Francesco Habetswallner, Rosa Iodice, Maurizio Marra, Luigi Michele Pavone, Mario Quarantelli, Giuseppe Vitelli, Mariarosaria Santillo, Roberto Paternò

**Affiliations:** 1 PRINDEX S.r.l., Via Cuma 28, 80132 Naples, Italy; pgiulia68@yahoo.it; 2 Dipartimento di Medicina Clinica e Chirurgia, Università di Napoli Federico II, Via Pansini 5, 80131 Naples, Italy; giuliana.larosa@unina.it (G.L.R.); sere.capozzi@unina.it (S.C.); simona.damiano@unina.it (S.D.); maurizio.marra@unina.it (M.M.); vitelli.giuseppe96@gmail.com (G.V.); marsanti@unina.it (M.S.); 3 Dipartimento di Neuroscienze, Scienze Riproduttive ed Odontostomatologiche, Università di Napoli Federico II, Via Pansini 5, 80131 Napoli, Italy; emanuele.cassano@unina.it (E.C.); rosa.iodice@unina.it (R.I.); 4 UOC Neurofisiopatologia, AORN Antonio Cardarelli, Via Cardarelli 9, 80131 Naples, Italy; francesco.habetswallner@aocardarelli.it; 5 Dipartimento di Medicina Molecolare e Biotecnologie Mediche, Università di Napoli Federico II, Via Pansini 5, 80131 Naples, Italy; luigimichele.pavone@unina.it; 6 Biostructure and Bioimaging Institute, Consiglio Nazionale delle Ricerche (CNR), Via De Amicis 95, 80145 Naples, Italy; quarante@unina.it

**Keywords:** multiple sclerosis, 5-HT2A receptor, diagnosis, blood serum, ELISA assay, peptides

## Abstract

Multiple sclerosis (MS) is an autoimmune chronic disease characterized by inflammation and demyelination of the central nervous system (CNS). Despite numerous studies conducted, valid biomarkers enabling a definitive diagnosis of MS are not yet available. The aim of our study was to identify a marker from a blood sample to ease the diagnosis of MS. In this study, since there is evidence connecting the serotonin pathway to MS, we used an ELISA (Enzyme-Linked Immunosorbent Assay) to detect serum MS-specific auto-antibodies (auto-Ab) against the extracellular loop 1 (ECL-1) of the 5-hydroxytryptamine (5-HT) receptor subtype 2A (5-HT2A). We utilized an ELISA format employing poly-D-lysine as a pre-coating agent. The binding of 208 serum samples from controls, both healthy and pathological, and of 104 serum samples from relapsing–remitting MS (RRMS) patients was tested. We observed that the serum-binding activity in control cohort sera, including those with autoimmune and neurological diseases, was ten times lower compared to the RRMS patient cohort (*p* = 1.2 × 10^−47^), with a sensitivity and a specificity of 98% and 100%, respectively. These results show that in the serum of patients with MS there are auto-Ab against the serotonin receptor type 2A which can be successfully used in the diagnosis of MS due to their high sensitivity and specificity.

## 1. Introduction

Multiple sclerosis (MS) is a chronic disease characterized by inflammation and demyelination of the central nervous system (CNS) associated with varying degrees of neuronal damage. It usually presents with recurrent, subacute, focal neurologic symptoms and signs that evolve into a relentless progressive decline of neurologic functioning, commonly affecting locomotion, bladder function, and cognitive skills [1]. 

A prompt diagnosis of MS allows for early therapeutic intervention, improving disease progression, reducing patient suffering, and lowering financial costs for both patients and the healthcare system [2,3]. The criteria for diagnosing MS have undergone multiple revisions, with the current McDonald criteria [4] relying heavily on magnetic resonance imaging (MRI). This diagnostic tool needs specialized training for interpretation, is often associated with high costs, and lacks optimal specificity. Clinicians following these criteria are reminded to consider alternative diagnoses. Additionally, the diagnosis requires an oligoclonal band test in the cerebrospinal fluid (CSF) sampling, obtained through an invasive procedure that requires a subjective interpretation. Hence, blood markers emerge as particularly attractive options for their ease of sampling; numerous biomarkers show promise for serving as diagnostic markers for MS. However, their utilization still has several limitations [5]. 

A substantial body of evidence supports the multifactorial etiology of MS. Besides genetic predisposition, several environmental risk factors have been associated with an increased risk of MS [6]. Among these, viruses are the microbial agents that have received the greatest attention for triggering or exacerbating multiple sclerosis. Most of the evidence in this regard concerns a role for the Epstein-Barr virus (EBV) in the development of MS [7,8,9,10], but the involvement of other viruses cannot be excluded.

Particularly intriguing is the role played by the JC polyomavirus (JCPyV) in the development of progressive multifocal leukoencephalopathy (PML), a neurodegenerative disease which targets oligodendrocytes (OLs) and astrocytes [11,12]. It has been shown that JCPyV requires serotonin receptors (5-HT2A, 5-HT2B, and 5-HT2C) to infect cells [13,14,15], and, most notably, that 5-HT2A receptor antagonists abrogate infection [13,16]. 

Serotonin has a well-established regulatory role in OL maturation, and serotonin pathway alteration leads to myelinating OL disfunction and, ultimately, to loss of myelin [17,18]. 

Some evidence links the 5-hydroxytryptamine (5-HT) pathway to MS. In relapsing–remitting MS patients (RRMS), there is a dysmetabolism of the serotonergic pathway. In the CSF of these patients [19], the levels of 5-hydroxyindoleacetic acid (5-HIA.A.), an index of serotonergic activity in the CNS, correlate negatively with disability accumulation during disease progression. Further, in experimental models of MS, like the one involving autoimmune encephalomyelitis (EAE) with variable demyelination levels, mice without the 5-HT transporter display a less severe disease progression compared to their wildtype counterparts [20]. Additionally, administering risperidone, an antagonist of the serotonin 5-HT receptor 2A, reduces both the size and quantity of spinal cord lesions in EAE-afflicted animals [21]. The expression of 5-HT receptor 1A and 2A subtypes has been demonstrated in the OL lineage [16,17], and the functional characteristics of the 5-HT2A receptor were demonstrated through the utilization of both agonist and antagonist [17]. 

Therefore, given the involvement of the serotonin pathway in MS and the use of the 5-HT receptor subtype 2A as a virus gateway to trigger a demyelinating disease, we speculated about the occurrence of the presence in MS sera of auto-antibodies (auto-Ab) against the 5-HT2A protein. For this purpose, we have developed an ELISA (Enzyme-Linked Immunosorbent Assay) test carried out with peptides belonging to the extracellular loop 1 (ECL-1) of the 5-HT2A receptor.

The results herein presented show that in the serum of patients with Relapsing–Remitting Multiple Sclerosis (RRMS) there are auto-antibodies against the serotonin receptor type 2A which can be successfully used in the diagnosis of multiple sclerosis due to their high sensitivity and specificity. 

## 2. Results

### 2.1. Peptide Designing

The 5-HT2A receptor belongs to the G-protein-coupled receptor super-family, with the characteristic presence of seven transmembrane domains [22]. A library was produced by Pepscan technology. Several hundred overlapping synthetic peptides for extracellular sequences were synthesized simultaneously on the heads of multiple polypropylene pins and used in solid-phase assays [23]. All the possible overlapping combinations were synthesized as linear as well as looped peptides, and the binding of 10 sera from control and 10 from MS patients were measured in an ELISA set-up. The binding of the different sera to all peptides was quantified and analyzed in detail. 

We first investigated the results obtained with linear peptides, and a list of the peptides with highest significant *p*-value of RRMS versus control sera (*p* < 0.05) was drawn up as shown in Table 1.

Since five out of seven peptides were localized on loop extracellular domain 1 (ECL-1), we focused on this region to design a peptide to use for the diagnosis of MS. An alignment of these peptides is shown in Table 2. For each one, the ECL-1 sequence is indicated, and we have highlighted in bold the positioning of the Pepscan peptide. From the alignment, we deduced a consensus sequence with the most represented amino acids. Therefore, we designed a peptide that was positioned centrally in the loop, away from the coiled coin and without amino acid substitution. The sequence of 12 amino acids (referred to as the LYG peptide from the first three amino acids of the peptide) is underlined (Table 2, bottom line) and was utilized in the subsequent binding experiments.

### 2.2. Set-up of ELISA for Detection of Auto-Antibodies in MS Patients

#### 2.2.1. Establishment and Optimization of Peptide-ELISA

**Figure 1:**
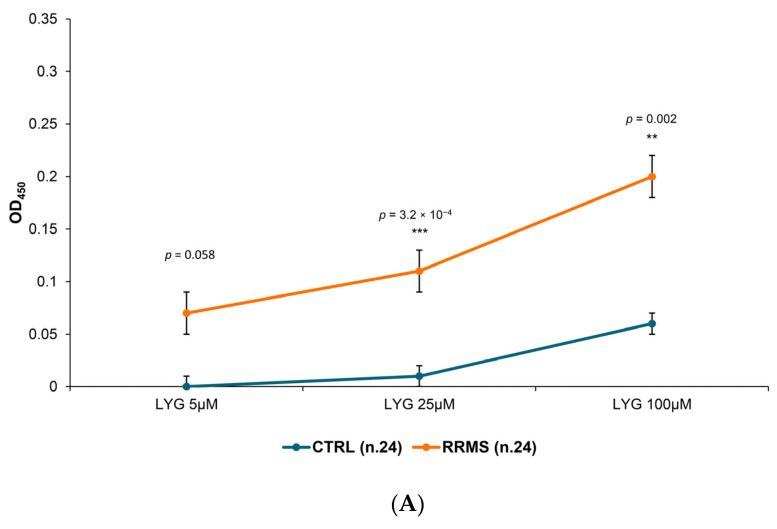

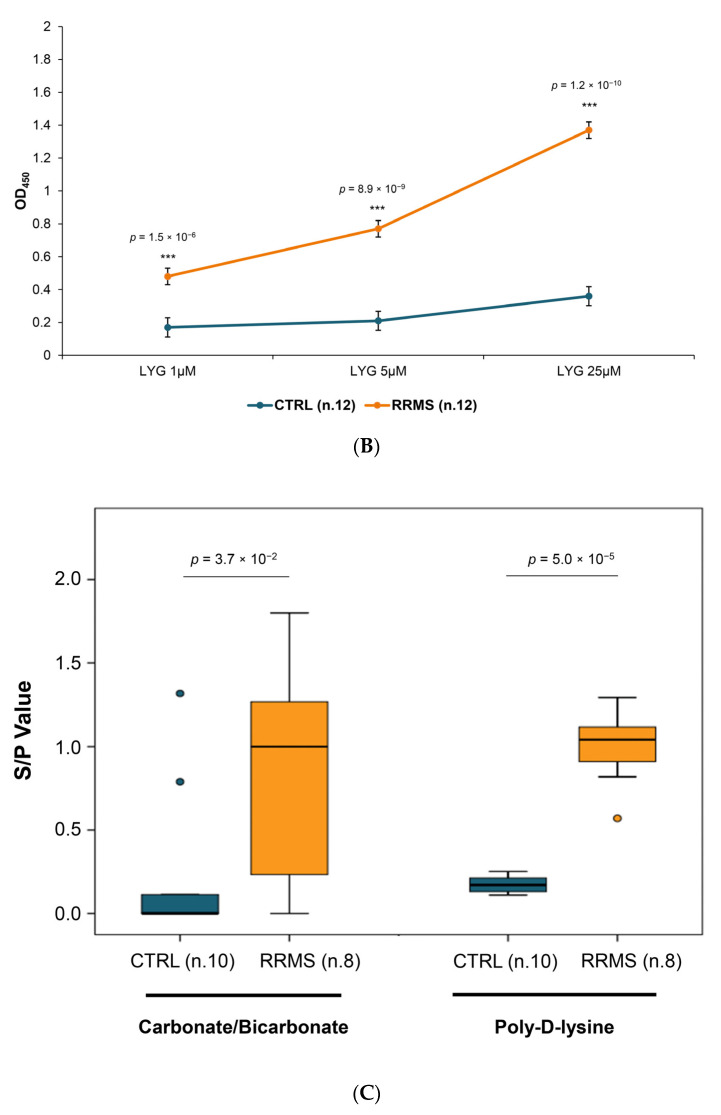
Comparison between coating in a carbonate/bicarbonate buffer versus poly-D-lysine. Dose-response curves on the two ELISA formats, performed with coating of a carbonate/bicarbonate buffer pH 9.6 (**A**) or with a previous pre-coat with 10 μg/mL of poly-D-lysine (**B**). The results are the mean ± SEM of *n* = 12 to 24 CTRL and RRMS sera performed in 3 independent experiments. The results are expressed as OD_450_ minus background. *p*-values were calculated with ANOVA (**C**). Intra-assay comparison of both ELISA formats on a panel of 10 CTRL and 8 RRMS sera in 3 independent experiments. The results are expressed as S/P values, as specified in Materials and Methods. *p*-values (calculated using the Mann–Whitney *t*-test) of significant differences are reported above the plot as asterisks. The asterisks ** and *** indicates, respectively, *p* ≤ 0.01 and ≤0.001.

In a peptides-based ELISA, a limiting step is the peptide immobilization on the plate. Initially, we coated the plates by diluting the LYG peptide in a 0.1 M carbonate/bicarbonate buffer (pH 9.6). However, the signal was notably low, even at the highest peptide concentration (RRMS mean OD_450_ ≤ 0.200; Figure 1A), despite a significant difference observed between RRMS patients and controls (CTRL) (*p*-value = 0.002). Following that, we investigated the possibility of amplifying the signal using a pre-coating agent, poly-D-lysine, a positively charged polymer known to enhance the adherence of peptides to the plastic [24,25,26]. We focused on lower concentrations, as poly-lysine enabled an approximately five times more efficient binding of the tested antigen than direct coating [26]. Figure 1B illustrates a titration curve of LYG concentration tested on a panel of RRMS versus CTRL sera on poly-D-lysine pre-coated microplates. The binding of RRMS sera increased dose-dependently compared to CTRL sera with both protocols (Figure 1A,B). However, the poly-D-lysine pre-coating significantly enhanced the OD_450_ signal, achieving the best positive/negative sample ratio at lower doses compared to the carbonate/bicarbonate buffer (25 μM versus 100 μM). 

Subsequently, we performed an intra-assay comparison between the two ELISA formats. The results, shown in Figure 1C and expressed as S/P values, as specified in the Materials and Methods section, indicated that sera tested on poly-D-lysine microplates yielded more reliable results than those tested on a carbonate/bicarbonate buffer (poly-D-lysine *p* = 5 × 10^−5^ versus carbonate/bicarbonate buffer *p* = 3.7 × 10^−2^). 

This suggests that poly-D-lysine pre-coating significantly improves the binding of our LYG peptide to plastic, allowing for a better discrimination between RRMS and CTRL specimens.

#### 2.2.2. ELISA Specificity

**Figure 2:**
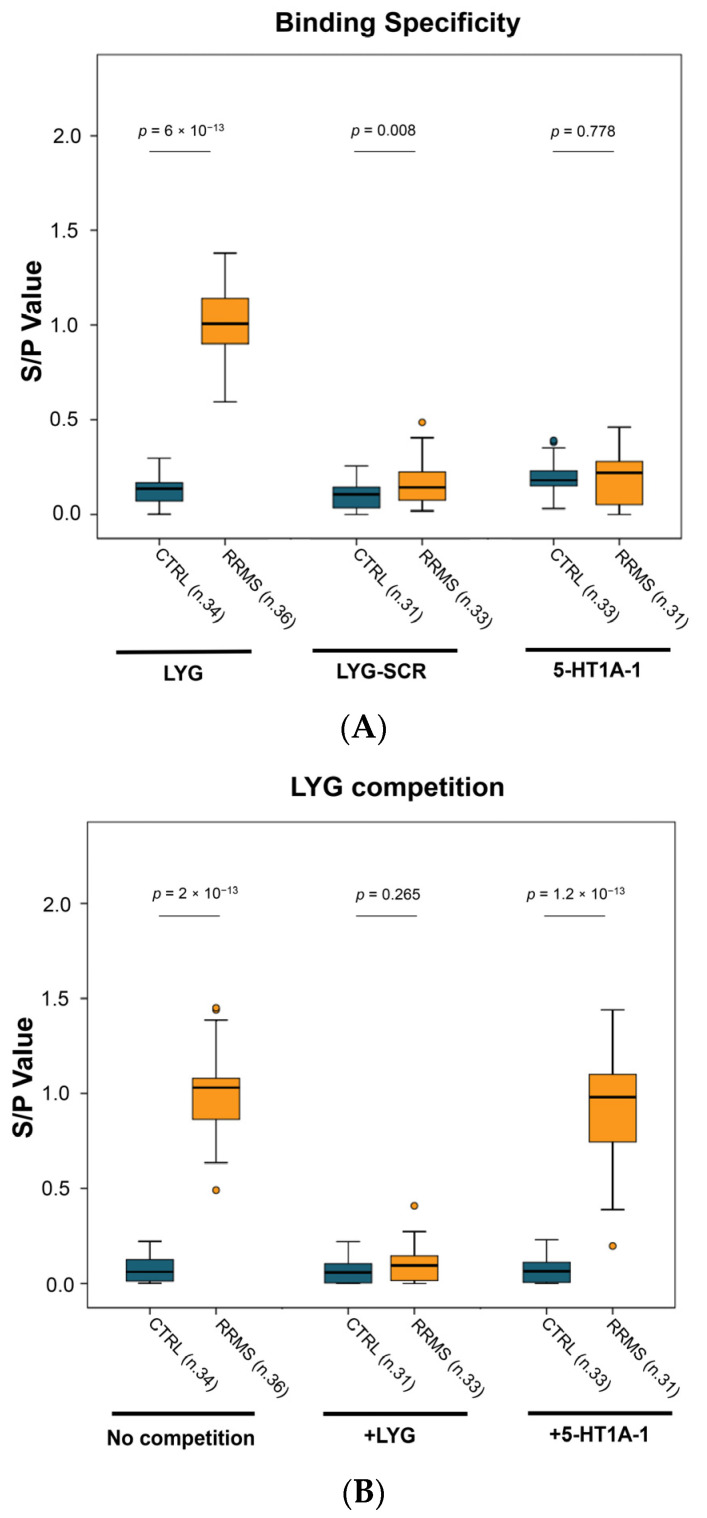
Analytical specificity on the poly-D-lysine ELISA format. Binding specificity on the poly-D-lysine ELISA format was assayed by (**A**) cross-reactivity on control peptides and (**B**) by competition experiments. (**A**) RRMS sera show binding activity only on the LYG peptide, whilst control peptides (LYG-SCR and 5-HT1A-1) did not react with RRMS sera. (**B**) Competition experiments were conducted by pre-incubation of sera with 5 μM of specific (+LYG) or non-specific (+5HT2A-1) peptide before plating on LYG-coated wells. The results are expressed as S/P values, as specified in Materials and Methods. More than 3 independent experiments were performed for each assay. All peptides were coated at 25 μM. RRMS and controls were compared using the Mann–Whitney *t*-test, and the *p*-values are reported above each plot.

We then assayed RRMS sera for cross-reactivity against control peptides. We used two different peptides, an LYG scrambled peptide (LYG-SCR) consisting of the same amino acids randomly positioned, and a peptide located in the extracellular domain 1 of a related 5-HT receptor family member, the 5-HT1A receptor (5-HT1A-1). As can be seen in Figure 2A, in comparison to the controls, the RRMS sera showed no binding to the 5-HT1A-1 peptide and low binding to LYG-SCR. Moreover, to verify the binding specificity on the poly-D-lysine pre-coated plates, we also performed competition experiments (Figure 2B). Sera from CTRL and RRMS subjects were pre-incubated with specific or non-specific peptide and then assayed for their residual binding activity on LYG-coated microplates. Pre-incubation of sera with the specific peptide (LYG itself) inhibited subsequent binding to the plate, whilst pre-incubation with 5-HT1A-1 was not able to compete with RRMS auto-Ab in respect to binding to the LYG sequence.

These results demonstrate that RRMS sera bind specifically to the ECL-1 domain of the 5-HT2A receptor.

#### 2.2.3. sIgG Binding

**Figure 3:**
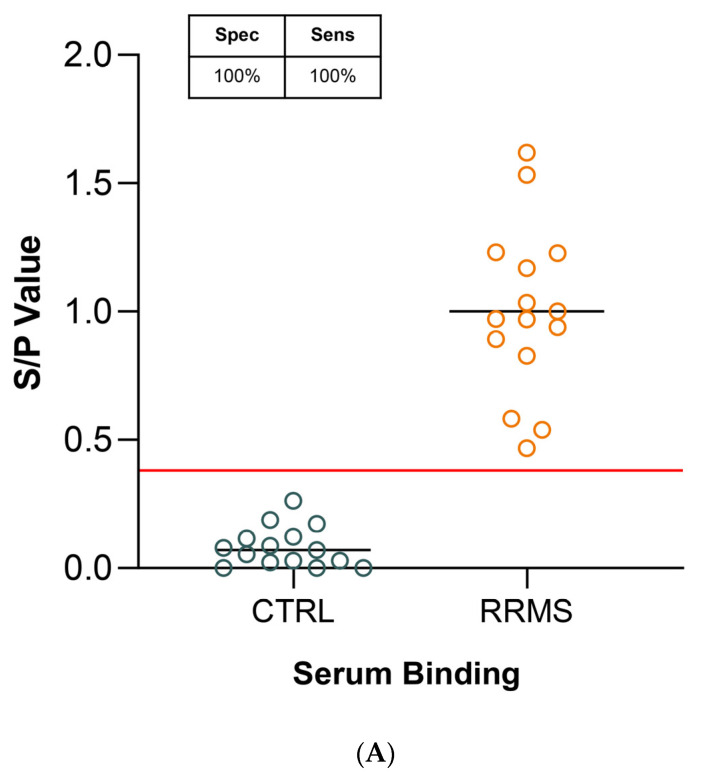

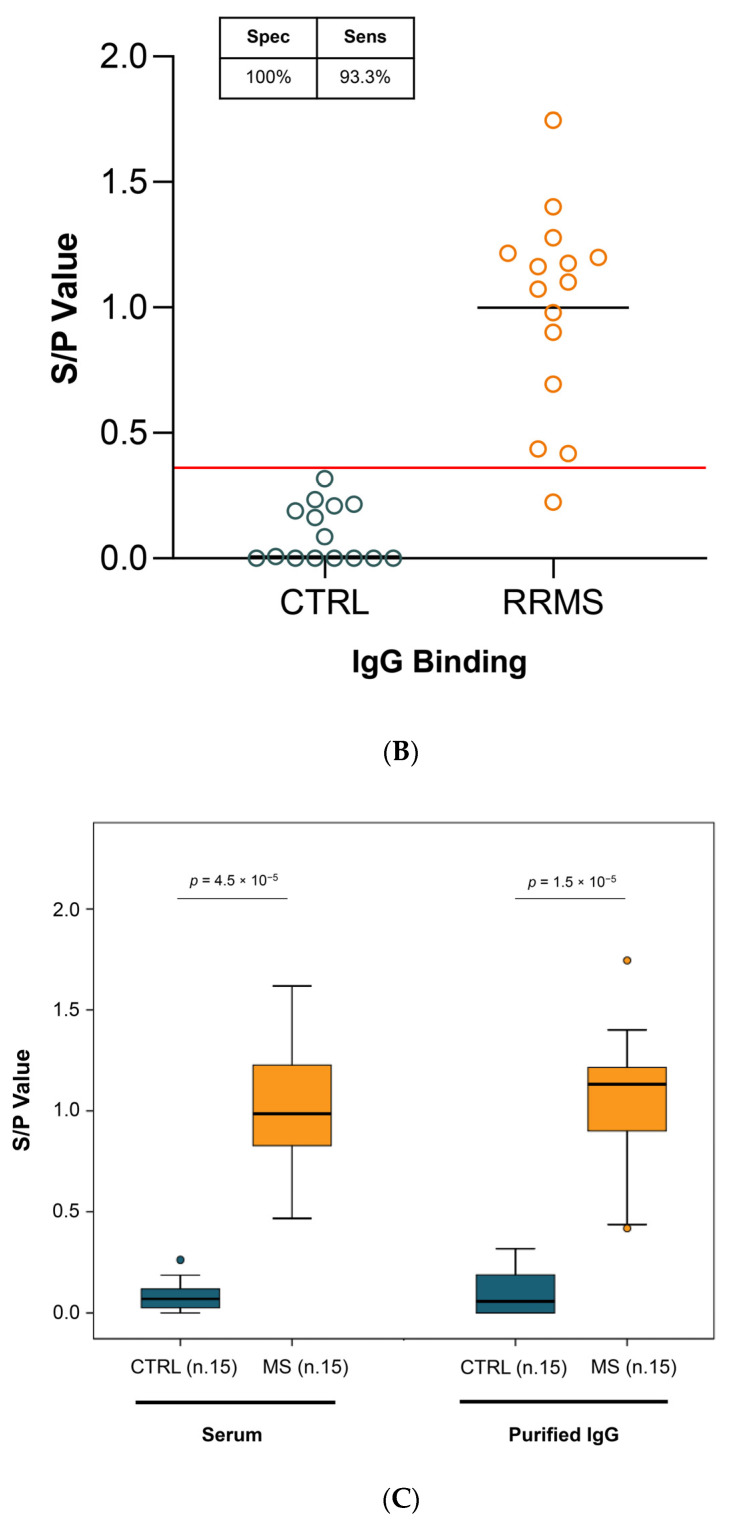
Serum compounds do not interfere with auto-Ab binding. IgG purified (10 μM) from a panel of 15 RRMS and 15 control samples was assayed in parallel with serum specimens (1:100 dilution) in 3 independent experiments. RRMS sera (**A**) and IgG (**B**) showed comparable binding activity on LYG. A horizontal red line indicated the cut-off value of 0.40 yielding the best sensitivity and specificity. RRMS and controls were compared using the Mann–Whitney *t*-test, and the *p*-values are reported above plot (**C**).

Next, we investigated the binding of IgG purified from the serum of RRMS patients to LYG peptide. Sera from 15 patients with RRMS and 15 healthy and/or pathological controls (systemic lupus erythematosus (LES), migraine, myasthenia, chronic inflammatory demyelinating polyneuropathy (CIDP)) were collected and their IgG were purified. Serum and purified IgG were tested in parallel in four independent experiments. IgG were tested at a concentration equivalent to sera dilution (10 μg and 1:100 dilution, respectively). As Figure 3A and 3B show, for both specimens (sera and purified IgG) the binding activity in RRMS samples was significantly higher compared to the controls, and the differences between RRMS and controls were comparable (Figure 3C; serum RRMS median 0.985, IQR 0.766–1.228 and serum CTRL median 0.07, IQR 0.022–0.122; IgG RRMS median 1.131, IQR 0.848–1.23 and IgG CTRL median 0.057, IQR 0.000–0.057).

Taken together, these data show that sera from RRMS patients contain auto-Ab that are able to specifically bind a peptide located in the ECL-1 domain of the 5-HT receptor subtype 2A.

### 2.3. Diagnostic Sensitivity and Specificity

The clinical and demographic characteristics of all participants in the study are presented in Table 3. Statistical analyses revealed no significant differences in gender and age distribution between the RRMS group and the control group, which included healthy subjects, patients without neurological diseases, and patients with neurological pathologies. The RRMS patient cohort included individuals with an average disease duration of 9.5 ± 8.5 years and relatively low average Expanded Disability Status Scale (EDSS) values (2.5 ± 9) (Table 3). At the time of blood collection, 93 (89.4%) patients were being treated with DMTs (mean treatment duration: 8.01 y ± 7.4) and 85% were relapse-free since DMT was started.

**Figure 4:**
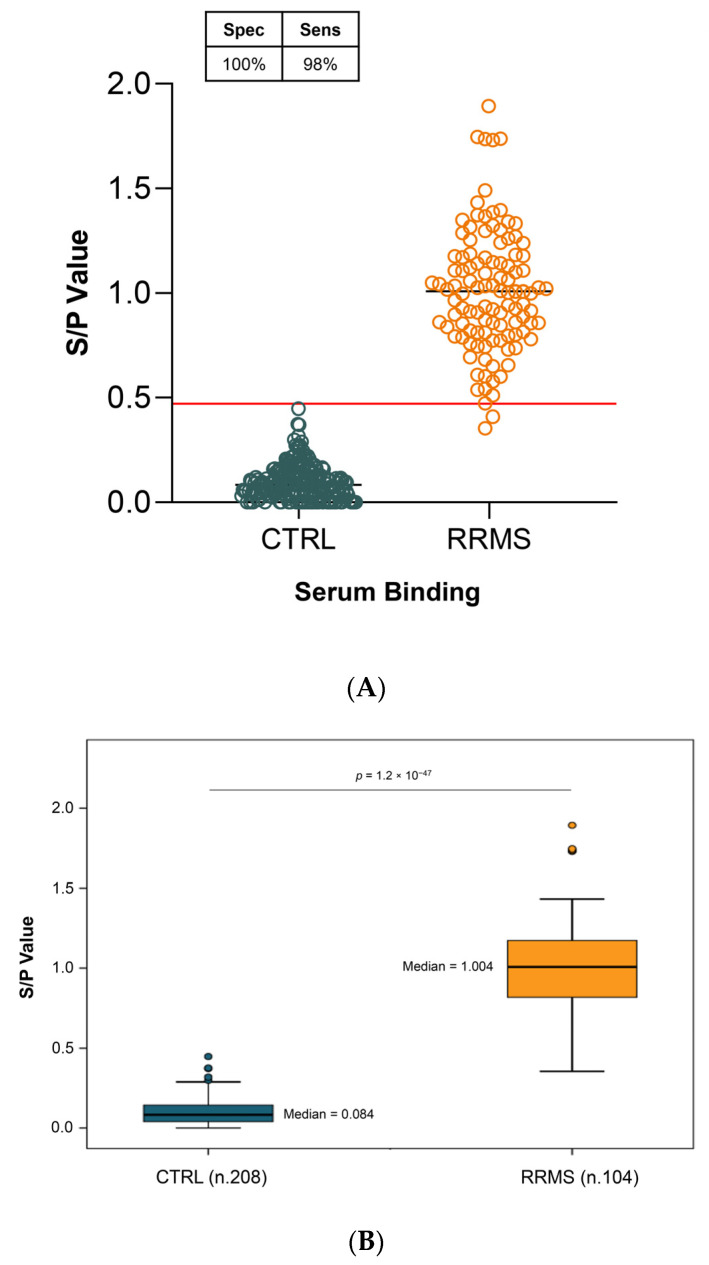
Serum autoantibody reactivity against LYG peptide. An ELISA assay was used to detect anti-5-HT2A serum auto-Ab. LYG peptide was coated on poly-D-lysine pre-coated microplates (**A**) The dot plot represents S/P values in 208 controls and 104 RRMS cases. A horizontal red line indicated the cut-off value of 0.48 yielding the best sensitivity and specificity (98% and 100%, respectively). (**B**) Box-and-whisker plots showing the distribution of S/P values in RRMS (median 1.004; IQR 0.814–1.172) and CTRL (median 0.084; IQR 0.040–0.143). RRMS and CTRL samples were compared using the Mann–Whitney *t*-test, and the *p*-values are reported above the plot.

ELISA experiments were conducted, and the results revealed a statistically significant difference in serum-binding S/P values between the 208 CTRL subjects and the 104 patients with RRMS (*p* = 1.2 × 10^−47^). Setting a cut-off value of 0.48 yielded a sensitivity of 98% and a specificity of 100% (Figure 4A). Statistical tests for pairwise comparisons revealed a more than tenfold decrease of S/P value in CTRL (median 0.084; IQR 0.040–0.143) subjects compared to patients with RRMS (median 1.004; IQR 0.814–1.172) (Figure 4B).

**Figure 5:**
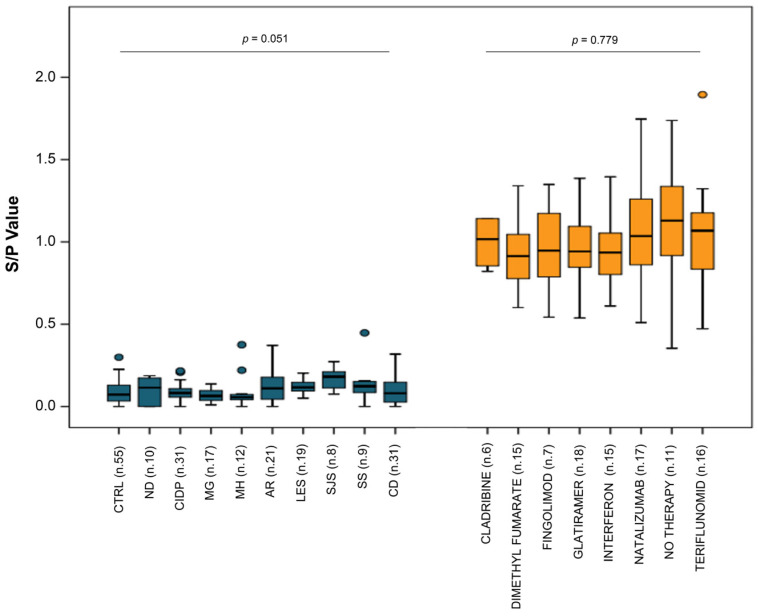
Autoimmune/neurological diseases and Disease-Modifying Treatment (DMT) do not affect LYG binding: the same data as used in Figure 4 are represented in the subgroups that made up each MS and control population. The statistical comparison between the subgroups (using the Kruskal–Wallis rank–sum test) revealed no significant differences within each cohort (control *p* = 0.051 for 10 groups and MS *p* = 0.779 for 8 groups). CTRL: healthy controls; ND: Neurologic Diseases; CIDP: Chronic Inflammatory Demyelinating Polyneuropathy; MG: Myasthenia Gravis; MH: Migraine Headache; AR: Rheumatoid Arthritis; LES: Lupus Erythematosus; SJS: Sjogren; SS: Systemic Sclerosis; CD: Coeliachia Disease.

We then conducted additional statistical analysis on both the control and RRMS patient populations to examine whether our test could effectively differentiate between RRMS patients and other pathologies. Furthermore, we explored whether the administration of different Disease-Modifying Therapies (DMTs) could interfere with the detection of our marker. As indicated in detail in Figure 5, serum-binding S/P value was analyzed in individual subgroups. Statistical tests for pairwise comparisons revealed that the serum-binding S/P values in the CTRL group were significantly lower than in all the RRMS groups. Moreover, no significant differences were observed either among subgroups of control patients or between subgroups of patients treated with Disease Modifying Therapies (DMTs) and those left untreated. 

Taken together, these data suggest that our ELISA test, performed with a peptide located in the extracellular loop 1 (ECL-1) domain of the 5-HT2A receptor, is able to accurately distinguish between positive and negative samples within the tested population.

## 3. Discussion

The major finding in the present study is that our ELISA test, using a simple blood sample, can detect the presence of MS specific auto-Ab, with a sensitivity and specificity above 95%. Moreover, being an accurate, inexpensive, and non-invasive test, it represents an excellent diagnostic alternative to the search for oligoclonal bands in the CSF.

To date, the laboratory tests commercially available for the diagnosis of multiple sclerosis include the measurement of Free Kappa Light Chains (kFLC) [27] and the quantification of Neurofilament Light Chains (NfL) and Glial Fibrillary Acid Protein (GFAP) levels [28,29,30,31,32,33]. The kFLC assay exhibits good sensitivity and specificity (78% and 87%, respectively) but it is performed on cerebrospinal fluid (CSF). Tests for NfL and GFAP levels are accessible through patients’ serum but are mainly of prognostic value. The levels of NfL and GFAP tend to increase in diverse conditions of axonal stress, making them more suitable for monitoring disease progression rather than serving as diagnostic tools. As a result of these limitations, despite being commercially available, these laboratory tests have not gained widespread adoption in clinical practice for diagnosing MS.

To our knowledge, our study is the first to report the possibility of making a diagnosis of MS with high sensitivity and high specificity using just a blood sample. The results disclosed in this manuscript were acquired through in-house assays, and additional steps are required before achieving a fully validated diagnostic product. The possibility of utilizing the latest diagnostic platforms is currently not feasible for our marker, as we do not have data regarding the specific amino acid residues involved in the antigen–autoantibody binding. Notably, under our specific conditions, passive coating of peptides has emerged as the most successful strategy, as mentioned below.

Multiple sclerosis is an autoimmune disease, and numerous studies have addressed the identification of the target antigens of patients’ auto-antibodies. A role for anti-KIR4.1 antibodies as a potential diagnostic marker has been investigated, but with conflicting results [34,35,36,37]. More recently, antigen microarrays have been shown to be useful for auto-antibodies profiling. A high-throughput platform built with continuous stretches of 80–100 amino acids residues as ‘bait’ allowed the identification of a set of 51 autoantigens potentially targeted by multiple sclerosis auto-antibodies [38,39,40].

Unlike the mentioned studies, our research strategy to identify auto-antigens in MS patients started from oligodendrocytes, the primary cellular target in demyelinating diseases such as MS and PML. These cells express members of the 5-HT2 receptor family, which serve as the gateway for JCPyV to induce injury and death in OLs [11,12,13,14,15,16,41]. Numerous pieces of evidence connect the serotonin pathway to MS [19,20,21], and a specific role for the 5-HT2A receptor has been demonstrated using agonist and antagonist molecules [16,17]. 

Then, to find putative MS auto-antigens, we conducted a comprehensive screening of all extracellular domains of the 5-HT2A receptor (specifically the N-terminal domain and the three extracellular loops) using a Pepscan microarray constructed with linear and looped peptides ranging from only 10 to 30 amino acids in length.

Our diagnostic test was focused on extracellular domain 1 (ECL-1) of the 5-HT2A receptor. Among the linear peptides with the highest significance, five out of seven mapped to extracellular domain 1 (ECL-1), and two to the N-terminal domain (Table 1). We investigated RRMS auto-Ab binding to both ECL-1 and the N-terminal domain. To this end, we designed a 12-amino acid peptide mapping on loop 1, along with three different peptides spanning the length of the N-terminal region (Table S1). Sera from RRMS patients bound the peptide located in the ECL-1 domain (LYG peptide) more efficiently than any of the three peptides located in the N-terminal domain (Table S1 and Figure S1), thus indicating that, among the peptides tested by Pepscan, the LYG peptide is the best diagnostic marker. The potential involvement of additional extracellular regions cannot be ruled out, and further studies will be necessary to comprehensively explore the 5-HT2A epitopes in patients with multiple sclerosis. However, it is noteworthy that another peptide located in ECL-1 of a related 5-HT2A receptor (namely, 5-HT1A receptor), also expressed in the OL lineage [17], does not exhibit reactivity towards MS sera (Figure 2A,B).

ELISA (Enzyme-Linked Immunosorbent Assay) is the current standard test for auto-Ab diagnostics worldwide due to its feasibility, sensitivity, and scalability. Various ELISA approaches for detecting antibodies exist [42]. In many cases, auto-Ab targets are protein fragments, but isolating, purifying, and immobilizing these antigens on solid supports in their native conformation pose challenges. Synthetic polypeptides that specifically bind to target antibodies are valuable ligands in antibody assays for their relative stability and the ease with which they can be synthesized. However, the binding of peptides to microplates depends on the length and charge of the synthesized peptides [43] and can be a limiting step in many ELISA peptide assays. Successful strategies to overcome this limitation include the use of functionalized peptides, such as GST, biotin, and digoxigenin fusion peptides [44], or the utilization of functionally activated microplates for the covalent attachment of peptides (Thermo Fisher Scientific Rockford, IL, USA). These approaches ensure a robust binding of the peptide to the plate. Nevertheless, in the absence of information about the epitope recognized by antibodies, it cannot be excluded that the covalent attachment of the peptide to the plate may interfere with the antigen–antibody interaction, particularly if the same amino acid residues are involved.

Given these considerations, we opted to pursue the path of passive coating, a method still recommended in recent literature as an effective coating approach for peptides [45,46]. We believe that in passive coating, random adsorption may more accurately replicate the native conformation of the epitopes involved in the antigen–antibody binding. To achieve this, we utilized a highly adhesive molecule, poly-D-lysine, as a pre-coating agent to enhance the binding of our peptide, which, in a buffer with a pH of 9.6, exhibited a very low (albeit specific) signal to MS auto-antibodies (Figure 1A,B).

The use of poly-D-lysine as a capture agent enabled us to develop an ELISA assay distinguishing sera from RRMS patients and controls with a high degree of significance (Figure 1C and Figure 4B). Furthermore, this binding is specific to the presence of antibodies, as purified IgG bound to the LYG peptide comparable to serum samples, with statistically similar values (serum *p* = 4.5 × 10^−5^ and IgG *p* = 1.5 × 10^−5^; Figure 3). Only a slightly lower sensitivity was observed in IgG specimens compared to sera (93% versus 100%). We hypothesize that this discrepancy could be attributed to the extraction method, which may not guarantee 100% recovery of serum IgG. 

This finding reinforces the utility of our test, as it can be performed on serum samples without requiring additional purification steps.

The results presented in Figure 4 and Figure 5 represent a diagnostic approach characterized by its ability to identify auto-antibodies in blood with remarkable sensitivity and specificity. The outstanding performance of the blood test we detailed is especially crucial in light of the misdiagnosis challenge. A survey among multiple sclerosis specialist neurologists in the USA revealed that 95% of respondents had encountered one or more patients in the preceding year who had received a misdiagnosis of multiple sclerosis. Many of these individuals were being inappropriately treated with disease-modifying therapies [1].

Given the great accuracy of this test in diagnosing the disease, it represents an excellent diagnostic alternative to the search for oligoclonal bands in the CSF. Furthermore, the presence of oligoclonal bands in the liquor is typical, but not exclusive to MS, and thus it can also be found in the course of other autoimmune, infectious, cerebrovascular diseases. In contrast, 5–10% of people with MS have a negative oligoclonal band test [5]. The blood test we have described can be complementarily employed alongside clinical examinations and instrumental diagnostics such as MRI and evoked potentials. 

This integration has the potential to streamline and enhance the treatment approach for MS. With a confirmed diagnosis of MS, drug treatment can be initiated. It is important to note that the drugs currently employed are mainly symptomatic and aim to decelerate the progression of the disease. Achieving an early and accurate diagnosis of multiple sclerosis is crucial, as numerous studies have demonstrated that initiating therapy with Disease-Modifying Therapies (DMTs) at an early stage significantly reduces the frequency and severity of relapses in MS patients compared to delayed treatment [5].

We applied our method to ten subgroups of patients within the control group, encompassing both healthy subjects and individuals with autoimmune or neurological diseases, some of which present a differential diagnosis with MS. Figure 5 illustrates that none of the ten control subgroups exhibited serum-specific binding to LYG, in contrast to all eight subgroups of patients within the RRMS group, whatever treatment they were undergoing. These results provide two crucial pieces of information: (i) they indicate that this test is unaffected by the type of therapy administered, and (ii) they suggest its potential capability to discriminate not only between RRMS patients and healthy individuals but also between RRMS patients and those affected by other conditions that may be part of the differential diagnosis with MS (MS mimics). Further and more extensive studies on larger population groups are necessary to confirm these data. 

Ongoing experiments aim to ascertain the earliest possible stage for MS diagnosis using this method. The investigation also extends to determining its applicability in diagnosing other forms of MS, such as secondary progressive and primary progressive MS, and assessing its potential in monitoring therapeutic effectiveness. Additionally, characterizing the epitope of the 5-HT2A receptor using this method could significantly contribute to identifying and understanding the sequence of an antibody with a potentially pivotal etiological role in the onset of MS. 

## 4. Materials and Methods

### 4.1. Patients and Healthy Controls

The inclusion of 104 patients was established on the basis of McDonald 2017 diagnostic criteria. The control group consisted of 55 healthy subjects and 153 pathological controls, comprising 78 subjects with no neurological diseases and 75 subjects with neurological pathologies. The subjects enrolled were selected from the Internal Medicine Department and the Neurology Department of the University “Federico II” di Napoli, Italy. Participants’ demographic and clinical data were obtained from hospital records or using a questionnaire.

### 4.2. Serum Sample Collection and Immunoglobulin Purification

Peripheral blood samples were collected in Vacutainer tubes (BD, SSTTM II Advance Plus Blood Collection Tubes) containing silica particles. The samples were centrifuged at 2000 rpm for 15 min at room temperature (RT) without brake to obtain serum. 

Immunoglobulin G (IgG) was isolated from the serum sample by immunoaffinity chromatography. First, 500 μL of serum was diluted 1:1 with phosphate-buffered saline (PBS) and the sample was run on a chromatography column with a solid phase consisting of A/G PLUS-Agarose resin (Pierce^TM^ Protein A/G Agarose-Thermo Fisher Scientific Rockford, IL, USA). The eluate was collected and the step was repeated three times. After several washes in PBS, 1 mL of Elution Buffer (Pierce IgG Elution Buffer- Thermo Fisher Scientific Rockford, IL, USA) was added to the chromatography column (six times) and six fractions were collected; 100 μL of Neutralization Buffer (1 M Tris pH 7.5–9) was added immediately. Absorbance was measured at 280 nm in a quartz cuvette with the spectrophotometer.

Subsequently, 1.25 mL of sample (obtained using 1 mL of the most concentrated sample and 0.250 mL of the second most concentrated) was loaded onto desalting columns (Pierce Dextran Desalting Columns—Thermo Fisher Scientific, Rockford, IL, USA). Six fractions were thus obtained. Absorbance was measured at 280 nm with the spectrophotometer and the three most concentrated samples were quantified by Bradford assay (Protein Assay Dye Reagent Concentrate-Biorad, Feldkirchen, Germany).

### 4.3. PEPSCAN-Based ELISA

The 455-well polypropylene cards containing the covalently linked peptides were incubated with a primary antibody solution consisting of diluted serum in a blocking solution (PBS with 1% Tween-20 and 4% horse serum). After washing, the cards were incubated with a 1:1000 dilution of antibody peroxidase conjugate for one hour at 25 °C. After washing, the peroxidase substrate ABTS and 2 μL/mL 3% H_2_O_2_ were added. After one hour, the color development was measured and quantified with a charge-coupled device (CCD) camera and an image processing system [47]. 

### 4.4. Synthetic Peptide Designing

Sequences of human 5-HT2A and 5-HT1A receptors were retrieved from the UniProt database (accession n° P28223 and P08908, respectively). To select the peptides, the sequences were aligned with the BLAST program, and peptides located in the ECL-1 were synthesized (LYG: LYGYRWPLPSKL; LYG-SCR: SLYPGKYRLPWL; 5HT1A-1: LNKWTLGQVT).

### 4.5. Peptide ELISA Assay

ELISA experiments were performed as follows. For the carbonate/bicarbonate protocol, 96-well ELISA plates (Nunc-Immuno module F8 Maxisorp—Thermo Fisher Scientific, Roskilde, Denmark) were coated with 100 μL of the synthetic peptide dissolved in 0.1 M carbonate/bicarbonate buffer (pH 9.6) and incubated at 4 °C overnight. After a 3 × 200 μL wash with PBS pH 7.4 containing 0.05% Tween-20 (PBS-T), the plate was blocked with 200 μL/well BB buffer (Pierce^TM^ Protein-Free Blocking Buffer—Thermo Fisher Scientific, Rockford, IL, USA) at RT for 1 h, and then 100 μL/well of serum samples (1:100 in BB buffer) were added and incubated at room temperature for an additional 2 h. After washes with the BBT buffer (Pierce^TM^ Protein-Free T20 Blocking Buffer—Thermo Fisher Scientific, Rockford, IL, USA), 100 μL/well of goat anti-human HRP-conjugated antibody (cat. no. 109-035-008, Jackson ImmunoResearch Europe Ltd.—Baltimore Pike, West grove, PA, USA), 1:5000 in BBT, was added as the secondary antibody and incubated at room temperature for 1 h.

The procedure for poly-D-lysine protocol is a modification of the assay described by Stearns et al., 2016 [26]. Briefly, 100 μL of 10 μg/mL poly-D-lysine hydrobromide (SIGMA cat. no. P7404) in PBS was added to Nunc-Immuno module F8 Maxisorp and incubated overnight at 4 °C. After 3 × 200 μL washes with cold PBS, 100 μL/well of peptide diluted in ELISA dilution buffer (PBS with 0.1 % BSA and 0.05% Tween-20) was added to the wells, and the plates were incubated 1 h at RT. After 3 × 200 μL washes with cold PBS, wells were blocked with blocking buffer (PBS with 2% BSA and 0.05% Tween-20) for 2 h at room temperature. Then, 100 μL/well of serum samples (1:100 in ELISA dilution buffer) were added and incubated at room temperature for 2 h. After 3 × 200 μL washes with cold PBS, 100 μL/well of goat anti-human HRP-conjugated antibody (1:5000 in ELISA dilution buffer) was added and incubated room temperature for 1 h.

For both protocols, color development was performed by adding 100 μL/well of TMB substrate (BioFX TMB One Component HRP Microwell Substrate—Surmodics, Eden Prairie, MN, USA) in the dark at 37 °C for 5–10 min, and the reaction was stopped using 50 μL of 500 mM H_2_SO_4_. The OD_450_ values were read with a plate reader, and OD_450_ of background (plastic absorbance) was subtracted from total OD values for each well. 

In each experiment, results were interpreted as S/P values [45] calculated according to the following formula:

S/P values = OD_450_ of sample/mean of OD_450_ of MS

Concerning ELISA specificity assay, S/P values reported for control peptides and for competition (collectively called “control tests”) were calculated as follows:

OD_450_ LYG: OD control tests = S/P value LYG: S/P value control tests

### 4.6. Statistical Analysis

A database was created using Microsoft Office Excel 365. Statistical analysis was performed on parametric and non-parametric data. A non-parametric statistical analysis was performed in the groups that had a non-Gaussian distribution due to a low number. In cases in which the comparison was between large groups (>100), such as between CTRL and RRMS (Figure 5), the differences were also evaluated with parametric tests (*t*-test), and, in any case, the results were comparable. Parametric data are presented as mean ± SEM; non-parametric data as median and interquartile ranges (IQR). *p*-value was obtained using ANOVA for parametric data, and Mann–Whitney (in two groups) and Kruskal–Wallis (in multiple groups) for non-parametric distributions. A *p*-value < 0.05 was considered significant for both parametric and non-parametric data.

## 5. Conclusions

In this manuscript, we present findings that indicate the presence in the blood serum of MS patients of antibodies against the serotonin receptor type 2A which can be successfully used in the diagnosis of MS due to their high sensitivity and specificity.

The results presented here were obtained through homebrew assays performed with a simple, cost-effective, and easily scalable ELISA approach, particularly suitable for analyses on larger populations and replicable in any laboratory equipped with a microplate spectrophotometer. However, before achieving a fully validated diagnostic product, additional steps are required. Specifically, reproducibility, robustness, and long-term stability should be tested on a prototype built according to current regulations. Additionally, clinical validation on a larger population (such as that achievable through a multi-center study) could definitively represent a paradigm shift in the diagnosis of multiple sclerosis, replacing the need for cerebrospinal fluid sampling. 

## 6. Patents

Diagnosis and Therapy of Multiple Sclerosis. Release numbers: USA 10633427, 28-April-2020 and EPO 3109257, 6-January-2021.

## Figures and Tables

**Table 1:** List of the best Pepscan peptides: the best linear peptide sequences synthesized by Pepscan and assessed using sera from 10 RRMS subjects versus 10 CTRL subjects. The experiments were conducted in duplicate, yielding a statistically significant *p*-value (<0.05) calculated using Student’s *t*-test.

Peptide Localization	Sequences PEPSCAN	Mean RRMS	Mean CTRL	*p*-Value
ECL-1	LTILYGYRWPAASKL	653	322	0.020
ECL-1	YRWPLPSKL	566	286	0.027
ECL-1	LTILYGYRWPLPSKL	647	340	0.03
ECL-1	GYRWPLPSK	623	323	0.038
N-term	TVDSENRTNLAAEGC	587	348	0.043
ECL-1	TILYGYRWPLPSKLC	652	378	0.046
N-term	STTNSLMQLNAATRL	612	385	0.047

**Table 2:** Alignment of Pepscan peptides to ECL-1: the positioning of the peptides within the ECL-1 domain is indicated, with peptides synthesized by Pepscan highlighted in bold. Bottom line: sequence of the LYG peptide designed based on the alignment of the Pepscan sequences.

Sequence ECL-1	Sequences PEPSCAN
LTILYGYRWP**SKLCAV	LTILYGYRWPAASKL
LTILYG**YRWPLPSKL**CAV	YRWPLPSKL
**LTILYGYRWPLPSKL**CAV	LTILYGYRWPLPSKL
LTILY**GYRWPLPSKL**CAV	GYRWPLPSK
L**TILYGYRWPLPSKL**CAV	TILYGYRWPLPSKLC
LTI**LYGYRWPLPSKL**CAV	**Sequence PRINDEX**

**Table 3:** Study cohort demographic and clinical characteristics. Data are expressed as mean ± SD. EDSS, Expanded Disability Status Scale. RRMS, Relapsing–Remitting Multiple Sclerosis.

Study Cohort		
	RRMS	CTRL
Number (n°)	104	208
Females (n°; %)	69 (66.6%)	124 (59.7%)
Age (years ± SD)	39.5 ± 8	42 ± 14
Disease duration (years ± SD)	9.9 ± 8.5	
EDSS (mean ± SD)	2.7 ± 1.9	
Patients DMT treated	93 (89.4%)	
DMT duration (years ± SD)	8.01 ± 7.4	
Patients relapse-free from DMT treatment	85%	

## Data Availability

The data presented in this study are available on request from the corresponding author. The data are not publicly available due to privacy and ethical restrictions.

## Supplementary Materials

The supporting information can be downloaded at: https://www.mdpi.com/article/10.3390/ijms25031696/s1.
