# Supplementary material for: A Blood Test for the Diagnosis of Multiple Sclerosis
Source: Int J Mol Sci. 2024 Jan 30;25(3):1696. doi: 10.3390/ijms25031696 (PMC10855725; doi:10.3390/ijms25031696)
Supplement: Supplementary file 1 [file ijms-25-01696-s001.zip › ijms-2807864-supplementary.pdf]

Table S1. List of PRINDEX peptides-mapping on ECL-1 (LYG peptide) and on the N-terminal region (N-ter 1, N-ter 2, N-ter 3). The peptides were assessed using sera of n = 12 to 27 CTRL and RRMS sera in 3 independent experiments. The results, expressed as **S/P values**, as specified in Material and Methods, are mean ± SEM. p-values are calculated using Student's t-test.

			Mean		SEM		*p*-Value CTRL vs RRMS
**N° CTRL**	**N° RRMS**	**peptide**	**CTRL**	**RRMS**	**CTRL**	**RRMS**	
23	27	LYG	0.094	0.998	0.018	0.058	1.3E-19
14	17	N-ter 1	0.023	0.106	0.008	0.028	0.013
20	20	N-ter 2	0.112	0.289	0.021	0.051	0.003
14	12	N-ter 3	0.037	0.161	0.012	0.040	0.007

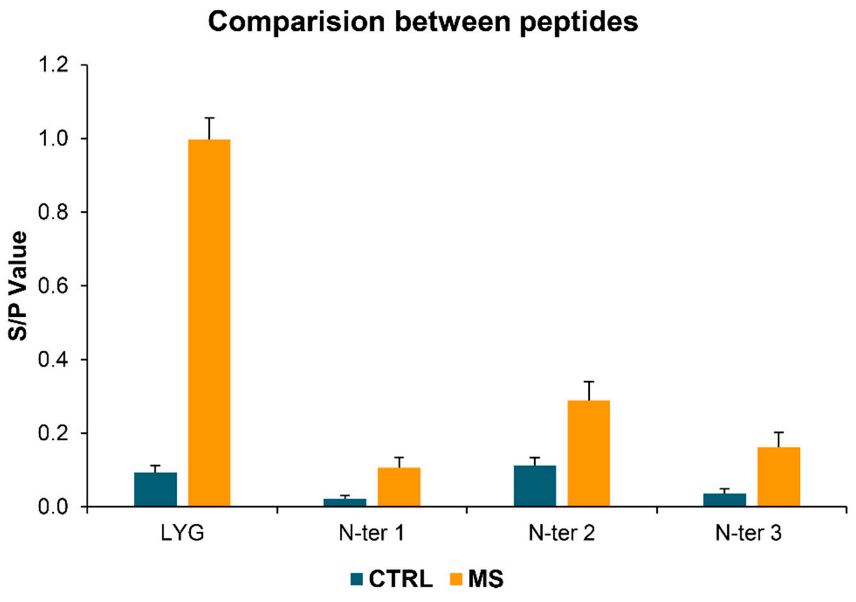

Figure S1. RRMS sera bind in the N-terminal region to a lesser extent. The bar plot shows the binding of n = 12 to 27 CTRL and RRMS sera to peptides mapping to ECL-1 and the N-terminal domain of the 5-HT2A receptor. The peptides were assessed in 3 independent experiments, and the results, expressed as **S/P values**, as specified in Material and Methods, are mean ± SEM.
